# Defining Patient and Public Involvement and Engagement Tasks in Health Data Research: A Consensus Study

**DOI:** 10.1111/hex.70578

**Published:** 2026-02-11

**Authors:** Hayley G. Evans, Olivia C. Robinson, Linda von Nerée, Brenda Smith, Rosamund Yu, Ester Bellavia, Jan Speechley, Julia Walsh, Simon Stanworth, Robbie Foy

**Affiliations:** ^1^ NIHR Blood and Transplant Research Unit in Data Driven Transfusion Practice, Radcliffe Department of Medicine University of Oxford Oxford UK; ^2^ Leeds Institute of Health Sciences University of Leeds Leeds UK; ^3^ NIHR UCLH Biomedical Research Centre London UK; ^4^ Health Data Research UK London UK; ^5^ Oxford University Hospitals NHS Foundation Trust John Radcliffe Hospital Oxford UK; ^6^ Warwick Medical School University of Warwick Warwick UK; ^7^ NHS Blood and Transplant John Radcliffe Hospital Oxford UK

**Keywords:** consensus methodology, data transparency, health data research, patient involvement, PPIE, public engagement

## Abstract

**Background:**

Patient and public involvement and engagement (PPIE) is essential throughout the research cycle, but a one‐size‐fits‐all approach does not suit all study types. Health data research presents several unique challenges, including ensuring data transparency and security. Thus, PPIE guidance should ideally define specific tasks to reduce the likelihood of tokenistic involvement. We therefore aimed to develop actionable, task‐focused guidance for PPIE in health data research.

**Methods:**

We used a consensus development process. We generated a list of potential PPIE tasks in health data research from existing literature informed by discussion with expert witnesses. We convened a consensus panel of nine members, comprising PPIE participants with varying experiences of PPIE and data research, PPIE professionals and health researchers. The panel first rated their agreement with tasks independently online and then discussed disagreements during an in‐person meeting, after which all tasks were re‐rated. We calculated median scores and refined a final set of tasks.

**Results:**

We identified 29 tasks across six domains of the research cycle: (1) prioritising and commissioning research; (2) planning research projects; (3) delivering research; (4) interpreting research; (5) sharing and using research knowledge; and (6) evaluating research. Consensus was reached on 25 tasks in the first round. Initial disagreements, particularly around planning research projects and monitoring data privacy, were resolved following structured discussions. Consensus and support were achieved for all 29 tasks following the second round.

**Conclusions:**

PPIE participants expressed a strong desire to be involved in all aspects of the research cycle. We offer a framework of actionable PPIE tasks for health data research and invite further development and evaluation.

**Patient and Public Contributions:**

PPIE participants were involved shaping the project idea and design, draughting a list of PPIE tasks, rating and discussing the importance of PPIE tasks and co‐writing the publication.

## 1 Introduction

Involvement of patients and the public in research is increasingly recognised as an essential component of good research practice. Meaningful patient and public involvement and engagement (PPIE) can enhance research relevance to community needs and priorities, improve study design, clarify study outcomes, and facilitate the integration of new evidence into practice [1, 2]. PPIE throughout all stages of the research cycle is now an established requirement for most UK public funding bodies (e.g. the National Institute for Health and Care Research; NIHR) [3, 4, 5, 6]. PPIE activities can include co‐designing grant applications, advising on study methods and protocols, developing participant‐facing materials, and contributing to dissemination [1]. However, a ‘one‐size‐fits‐all’ approach may not be appropriate for all types of research and PPIE expectations and contributions should be tailored to the research goals [7]. Additionally, despite its growing prominence, the legitimacy of PPIE has sometimes been questioned, especially when involvement appears tokenistic or limited to a ‘box‐ticking’ exercise [1, 8]. These risks may increase when PPIE roles are unclear or not linked to specific tasks that define contributions to the research process. This has led to the development of the UK standards for Public Involvement Frameworks [4] and guidance from charity organisations, funding bodies and research groups to support meaningful and inclusive PPIE. PPIE participants are the preferred term used throughout this document to refer to the diverse patient and public members involved in this work.

Health data research is a rapidly expanding and evolving field. It involves gathering, analysing, and linking information about people and their health to understand disease and improve healthcare [9]. Unlike traditional clinical research, which typically requires informed consent from participants, health data studies often rely on broad, public consent mechanisms such as ‘opt‐out’ or public interest approval presenting unique ethical and regulatory challenges [10]. These challenges include ensuring transparency, maintaining public trust, and addressing concerns around data privacy and security [11]. Building trust between researchers and people whose data is used, including patients and the public, has been emphasised as a cornerstone for responsible health data research [12, 13]. Mutual collaboration between researchers, patient and the public is required so that ethical considerations, such as data privacy and discussions around sharing health data, are addressed in a responsible and acceptable way [10, 12, 14, 15, 16, 17]. Engaging PPIE participants in health data research is crucial to inform and shape understanding of data access, consent mechanisms, and ethical safeguards, and is a way of ensuring that the research aligns with public values and concerns [18].

Whilst guidance on PPIE in health research is available [4, 19, 20], there is limited direction on incorporating PPIE effectively into health data research. Emerging health data strategies [21, 22] outline objectives for PPIE engagement, such as developing communications plans to promote trustworthiness and transparency around health data research and improving language and accessibility for stakeholders and under‐represented voices [21]. However, task‐specific recommendations on how to embed PPIE at each phase of the health data research cycle are lacking.

The Data‐Driven Blood and Transfusion Research Unit (DD‐BTRU) was established to accelerate the development of data‐driven methods to optimise blood use and integrate them within routine practice to improve patient outcomes [23]. The programme is structured around four interlinked work packages: (i) measuring inappropriate variations in transfusion practice and identifying data‐driven interventions to improve patient care, safety and value for money; (ii) developing novel data linkages and electronic systems to support transfusion pathways throughout the blood supply chain; (iii) establishing the infrastructure for scaled‐up clinical, epidemiological and quality improvement research using large data sets to inform policy and patient care and; (iv) modelling the cost‐effectiveness of resulting innovations. We were unaware of any task‐specific PPIE guidance for health data research. We therefore aimed to produce actionable guidance to support inclusion of PPIE in future health data research.

## 2 Methods

### 2.1 Design

**Figure 1:**
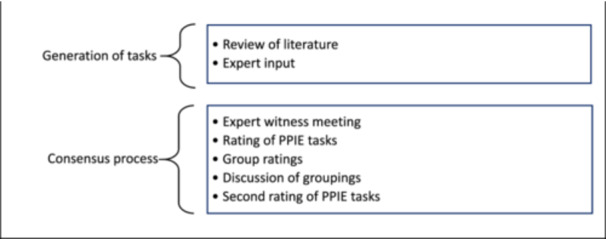
PPIE task generation and consensus process Figure 1. This diagram summarises the process of generating, refining and prioritising PPIE tasks in health data research. The process started with generating a list of PPIE tasks from literature and incorporating expert before using a consensus process to prioritise PPIE tasks aligned with six key phases of a research project.

We used a modified RAND consensus development process [24], a formal group consensus process that systematically and quantitatively combines expert opinion and evidence by asking panellists to rate, discuss, then re‐rate items (Figure 1).

We chose this over other methods (e.g. Delphi) as it allows structured deliberation of potentially complex issues and provides an explicit method of aggregating judgements.

### 2.2 Participants

We convened a panel of nine people associated with the DD‐BTRU. This included two researchers, two PPIE professionals and five self‐selected PPIE members from the DD‐BTRU Patient and Public Panel. Six panellists were female and three males. The panel reflected a range of ethnic backgrounds: two Asian or Asian British, three Black‐British, Black‐African or Black‐Caribbean, and four White‐British or Other. Self‐rated experience of PPIE in research was three experts, three intermediate and three beginners with varying levels of health data‐research experience. Panel membership was weighted towards patient and public members to ensure that their experiences and reflections formed the majority contribution. All patient and public members involved in this project received payment for their contributions PPI payment guidelines from the NIHR [25]. They could select between online vouchers of their choice or bank transfers. Whilst we use the term ‘PPIE participants’ throughout this paper, we acknowledge it encompasses both people with lived experience of specific health conditions or service settings and members of the public who contribute broader societal perspectives [26].

### 2.3 Generation of Tasks

**Figure 2:**
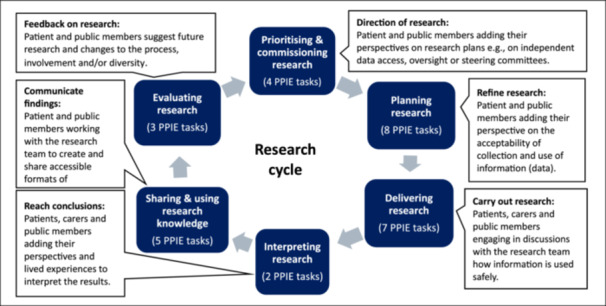
The distribution of PPIE tasks in health data research over the research cycle Figure 2. The distribution of PPIE tasks in health data research over the research cycle is shown alongside the six phases of the research cycle. These include prioritising and commissioning research; planning research projects; delivering research; interpreting research; sharing and using research knowledge; and evaluating research. The number of tasks identified in each phase is shown along with examples of the possible tasks.

We drew upon existing literature to generate a list of potential PPIE tasks in health data research [7, 27, 28, 29, 30]. We focused on specific tasks with defined actions (e.g., giving feedback on draft funding applications for research) rather than roles (e.g., data oversight committee membership) to be consistent with our goal of providing clear expectations. We shared and refined the list of tasks following feedback from researchers, patient and public members and PPIE managers including PPIE participants in the panel. We grouped the resulting tasks within six phases of the research cycle: prioritising and commissioning research; planning research projects; delivering research; interpreting research; sharing and using research knowledge; and evaluating research (Figure 2).

### 2.4 Consensus Process

We invited three ‘expert witnesses’ to share their perspectives and to familiarise PPIE participants with background issues in an optional 1‐h online meeting. The experts comprised a data researcher with PPIE experience, a PPIE manager and a PPIE partner with health data research experience. All five PPIE members and one PPIE Manager attended the meeting with ‘expert witnesses’. After this meeting, panellists independently rated PPIE tasks online using a 9‐point Likert scale (1=strongly disagree to 9=strongly agree). We aggregated panel ratings. High disagreement was defined as where at least two panellists ranked an item at the lower end of the scale (1–3) and at least two ranked it at the higher end of the scale (7–9). Moderate disagreement was defined as at least two panellists rating at either end of the scale. High or moderate disagreement indicated areas where consensus was not yet reached. The absence of high or moderate disagreement indicated consensus. We calculated median ratings for each PPIE task.

We shared aggregated ratings with all participants, and they were given a copy of their initial ratings for reference at the start of the 5‐h consensus meeting, which all nine panellists attended in‐person. We discussed disagreements with the goal to promote, but not force, consensus. Participants had the opportunity to share their thoughts on the importance of each task, clarify meaning and discuss reasons for their rating decision. During the consensus meeting participants ranked and submitted each PPIE task again after discussing it. The results of the consensus process were shared with participants to allow further reflections and feedback before we refined the selected tasks.

## 3 Patient and Public Contributions

One PPIE participant, from three invited, joined the project management team, attending all meetings and contributing to decisions on design, management, delivery, interpretation and dissemination. Eleven members from the DD‐BTRU Patient and Public Panel helped shape the project idea and design. Six PPIE participants draughted and rated a list of PPIE tasks, also discussing their importance. Two PPIE participants co‐wrote the publication. Seven PPIE participants were involved in developing a subsequent version of the PPIE tasks framework for patients and the public, as well as in writing and reviewing the manuscript.

## 4 Results

We identified a total of 29 PPIE tasks across the research cycle (Table 1). We also generated a version with examples to help understanding for panellists (Supporting Information Table S1). All panellists completed both rating processes and contributed to the meeting discussions.

**Table 1:** Patient and public involvement and engagement (PPIE) tasks identified across the research cycle.

**Prioritising and commissioning research (deciding what research should be done)**
Suggesting research topics
Setting research priorities for specific health conditions
Making recommendations about which research to fund
Give views on direction of research projects
**Planning research projects (working out how to do the research)**
Adding skills and experiences to applications for research funding
Help to shape research questions (e.g., adding questions patients see as important).
Helping decide how information is collected and analysed
Guiding the development of treatment and health care services
Voicing concerns about areas of the project and helping to find solutions
Advising the researchers about recruitment of participants for the study
Reviewing information to encourage trustworthiness and transparency
Advising the research team on their Patient and Public Involvement and Engagement plans
**Delivering research (carrying out the research)**
Helping to write the research materials
Reviewing documents about research made for patient and public members
Encouraging and supporting the involvement of people with diverse views
Supporting the delivery of Patient and Public Involvement and Engagement plans
Helping to build connections to patient networks and communities
Monitoring the protection of privacy and confidentiality of patient information
Reviewing the progress of research (e.g., getting regular updates about the work)
**Interpreting research (looking at the results of the research)**
Assisting the research team to interpret results and develop recommendation from them
Advising how a treatment or service can continue after the study
**Sharing and using research knowledge (telling people about the research)**
Sharing knowledge from a research study (e.g., helping write up the results)
Advising how to feedback results to research participants
Adding a personal perspective about the results
Speaking about the research results and point out groups to discuss it with
Give personal insights into how patient and public members see the results
**Evaluating research (reflecting on the research and how it was done)**
Contribute to the evaluation of the research project
Help to evaluate the impact of Patient and Public Involvement and Engagement
Make future research recommendations (e.g., suggesting future research questions)

*Note:* A total of 29 PPIE tasks were identified across the research cycle.

Results of the first rating process indicated consensus in support of 25 tasks (Table 2). We found moderate disagreement for three tasks within the research planning phase: helping decide how information is collected and analysed (median score 7; range 2–8), voicing concerns about areas of the project and helping to find solutions (8; 3–9) and reviewing information to encourage trustworthiness and transparency (8; 3–9). We found high disagreement for one task: monitoring the protection of privacy and confidentiality of patient information (7; 2–9) (Table 2).

**Table 2:** Consensus ratings of PPIE tasks.

Tasks	1st round scores			2nd round scores		
	Mean	Median	Range	Mean	Median	Range
1. Suggesting research topics	7.6	8.0	(5–9)	7.6	7.0	(5–9)
2. Setting research priorities for specific health conditions	7.6	7.5	(6–9)	8.0	8.0	(7–9)
3. Making recommendations about which research to fund	7.8	8.0	(6–9)	7.8	8.0	(6–9)
4. Give views on direction of research projects	7.5	7.5	(5–9)	7.6	7.0	(5–9)
5. Adding skills and experiences to applications for research funding (e.g., helping to write the application)	6.8	7.0	(4–9)	7.3	8.0	(5–9)
6. Help to shape research questions (e.g., adding questions patients see as important).	8.0	8.5	(6–9)	8.3	9.0	(6–9)
7. Helping decide how information is collected and analysed	6.7	7.0	(2–8)	7.6	7.0	(6–9)
8. Guiding the development of treatment and health care services	7.3	8.0	(4–9)	7.6	8.0	(6‐9)
9. Voicing concerns about areas of the project and helping to find solutions	7.6	8.0	(3–9)	7.9	8.0	(7–9)
10. Advising the researchers about recruitment of participants for the study	7.4	7.0	(5–9)	7.9	8.0	(6–9)
11. Reviewing information to encourage trustworthiness and transparency	7.4	8.0	(3–9)	7.6	8.0	(5–9)
12. Advising the research team on their Patient and Public Involvement and Engagement plans	8.0	8.0	(5–9)	8.0	8.0	(6–9)
13. Helping to write the research materials	6.8	7.0	(4–9)	7.4	8.0	(5–9)
14. Reviewing documents about research made for patient and public members	8.3	9.0	(7–9)	8.8	9.0	(8–9)
15. Encouraging and supporting the involvement of people with diverse views	8.4	9.0	(7–9)	8.6	9.0	(7–9)
16. Supporting the delivery of Patient and Public Involvement and Engagement plans	8.2	9.0	(6–9)	8.4	9.0	(7–9)
17. Helping to build connections to patient networks and communities	8.7	9.0	(7–9)	8.8	9.0	(8–9)
18. Monitoring the protection of privacy and confidentiality of patient information	5.9	7.0	(2–9)	6.8	7.0	(5–9)
19. Reviewing the progress of research (e.g., getting regular updates about the work).	7.2	7.0	(4–9)	7.3	7.0	(5–9)
20. Assisting the research team to interpret results and develop recommendation from them.	6.8	7.0	(5–9)	7.0	7.0	(5–9)
21. Advising how a treatment or service can continue after the study.	7.2	7.0	(5–9)	7.3	7.0	(5–9)
22. Sharing knowledge from a research study (e.g., helping write up the results).	7.3	7.0	(6–9)	7.7	7.0	(7–9)
23. Advising how to feedback results to research participants	7.8	8.0	(6–9)	8.0	8.0	(7–9)
24. Adding a personal perspective about the results	7.9	8.0	(5–9)	8.0	8.0	(7–9)
25. Speaking about the research results and point out groups to discuss it with	8.0	8.0	(7–9)	8.0	8.0	(7–9)
26. Give personal insights into how patient and public members see the results.	7.9	8.0	(6–9)	7.9	8.0	(6–9)
27. Contribute to the evaluation of the research project	7.7	8.0	(6–9)	8.1	8.0	(7–9)
28. Help to evaluate the impact of Patient and Public Involvement and Engagement	8.0	8.0	(6–9)	8.2	8.0	(6–9)
29. Make future research recommendations (e.g., suggesting future research questions).	7.6	7.5	(6–9)	8.1	8.0	(7–9)
		Minor disagreement				
		Major disagreement				

*Note:* Ratings of 29 Patient and Public Involvement and Engagement (PPIE) tasks across the research cycle. Shown are mean, median, and range scores from panel members for each task. Minor (moderate) and major (high) disagreements are highlighted in the table.

After a second round of rating and discussion consensus was reached for all 29 tasks (Table 2). The most strongly‐rated tasks included: reviewing documents about research made for patient and public members (9; 8–9); encouraging and supporting the involvement of people with diverse views (9; 7–9); supporting the delivery of PPIE (9; 7–9); and helping to build connections to patient networks and communities (9; 8–9).

## 5 Discussion

### 5.1 Summary of Findings

We identified and prioritised 29 PPIE tasks relevant to health data research. Through a structured consensus process, we established that there is strong agreement on the importance of these tasks across all phases of the research cycle. Our comprehensive framework also offers actionable guidance for researchers and a basis for developing and monitoring PPIE in health data research. The growth of health data research PPIE strategies [21, 22, 31] amplifies the importance of identifying specific PPIE tasks that can help operationalise PPIE involvement throughout the health data research cycle [12, 14].

Our approach and findings broadly mirror those of an earlier consensus study, which found strong support and consensus for 20 out of 21 roles of PPIE in clinical research [7]. That same study found more focused support for a narrower range number of roles in implementation research, which often focuses on understanding and changing the behaviour of healthcare organisations and professionals rather than patients. Our findings highlight that PPIE is desired throughout every stage of health data research, from prioritising and commissioning studies to evaluating their impact.

Our findings confirmed that trustworthiness, transparency and ethical oversight, key requirements for health data research, were also considered central to PPIE. Subsequently, rather than PPIE members being directly involved in shaping, delivering and testing interventions like other health research studies [7, 32], tasks were orientated towards governance, advising and interpretation of results to show trustworthiness. This may reflect the specific challenges of working with health data, where there are public concerns about potential misuse, consent, and data security [12, 14]. The tasks presented are a prompt for researchers to explore opportunities for involvement and engagement; they are not exhaustive and will need further refinement as research expectations and methods evolve. Whilst PPIE should remain flexible and iterative, defining specific tasks can help ensure that contributions are meaningful and likely to be acted upon [33].

Research planning tasks (e.g., deciding how information is collected and analysed) were initially associated with moderate or high disagreement, possibly reflecting the varying experience and skills of PPIE participants [33]. Differences in experience, and the support available to them, may influence how PPIE participants engage with technical aspects of study design and methodological discussions. Appropriate support structures can help maximise involvement [34]. PPIE can enhance clinical research planning by improving its relevance and quality, ensuring patient‐centred outcomes, and increasing the accessibility of findings [1]. Subsequently, if PPIE is desired at each stage of health data research, offering training and capacity‐building initiatives could support PPIE participants to provide meaningful input throughout all aspects of the research cycle [35, 36].

### 5.2 Study Strengths and Limitations

A key strength of our study concerned the use of a modified RAND consensus development process [24], which combines private, independent decision‐making with collective, face‐to‐face deliberations. This approach allowed for a more nuanced understanding of each PPIE task and facilitated a balanced evaluation, ensuring that diverse perspectives were considered. The relative diversity of our panel, encompassing varying levels of expertise and backgrounds, further contributed to the robustness of the findings. Discussions with PPIE participants can highlight additional PPIE priorities, including the need to engage underrepresented groups, such as minority communities, in conversations about the benefits, limitations, and transparency of data use to support trust and inclusivity.

A limitation is that our panel was derived from only one health data research programme addressing a specialist field‐blood and transplant. We recognised the risk that the two researchers on the panel may potentially have exerted disproportionate influence on group discussions. We attempted to mitigate this by structuring all deliberations and use of a formal consensus process. The identified tasks may also require refinement as health data research evolves. Future studies could benefit from larger, more diverse panels, including international participants, to validate and elaborate our findings.

### 5.3 Implications for Practice and Research

Our work complements existing PPIE tools and best practice guidelines, such as the UK Standards for Public Involvement in research and the Public Engagement in Data Research Initiative (PEDRI) [4, 37]. Our practical guidance aims to encourage meaningful PPIE, which can promote the ethical conduct, relevance, and transparency of health data research. For researchers, this means incorporating patient and public insights at all stages of data research, potentially leading to more patient‐centred outcomes. Future research should explore how the distinct differences between people with lived experience and the public might be aligned with certain tasks and stages of the research process. Both groups bring different but complementary perspectives to health data research; patients contribute experiential knowledge on research questions, methods and outcomes, whilst public can offer broader views on funding, social and ethical considerations [26]. However, our framework provides a foundation to help inform policymakers and funding bodies in clarifying requirements for PPIE in health data research proposals and evaluations.

If PPIE is desired throughout all stages of the research cycle, it is important to provide PPIE contributors with the skills and training needed for active and inclusive engagement [35, 36]. Future research should explore the types of support structures that enhance PPIE impacts and how experienced participants can mentor those new to PPIE to ensure a more inclusive and supportive environment.

PPIE should be viewed as an essential part of good research practice, therefore promoting mutual learning through evaluation can provide valuable insights into how PPIE can be organised and supported most effectively [38]. Previous literature has supported the use of PPIE evaluation at varied timepoints in the research cycle. This includes understanding research processes, context and outcomes to enhance learning and accountability [39]. In addition, diverse approaches to PPIE have been shown to enhance recruitment and retention in clinical trials [38]. Such evaluations determine what works, for whom and under what conditions, while recognising PPIE as a fundamental component of research. We therefore welcome further work to develop and evaluate our task‐oriented framework to guide research planning and monitoring of PPIE.

## 6 Conclusion

This study highlights that PPIE is important throughout a research project, but specific tasks can vary between different study types. Building on well‐defined PPIE tasks for clinical trials, this study started to define a list of specific PPIE tasks for health data research. The consensus process of this study found strong support for and agreement around PPIE tasks across six key phases of health data research. Findings and a list of specific PPIE tasks offer a framework to build on, guide and monitor PPIE in health data research, supporting researchers and patient and public members to better plan and implement meaningful involvement and engagement in health data research.

## Author Contributions

**Hayley G Evans:** conceptualisation, investigation, writing – original draft, methodology, validation, visualisation, writing – review and editing, formal analysis, data curation, supervision, resources. **Olivia C Robinson:** writing – original draft, writing – review and editing. **Linda von Nerée:** conceptualisation, investigation, writing – original draft, writing – review and editing, visualisation, project administration, resources, supervision. **Brenda Smith:** conceptualisation, investigation, writing – original draft, writing – review and editing, supervision. **Rosamund Yu:** investigation, funding acquisition, writing – original draft, writing – review and editing. **Ester Bellavia:** writing – original draft, writing – review and editing, investigation, validation. **Jan Speechley:** investigation, writing – original draft, writing – review and editing. **Julia Walsh:** investigation, writing – original draft, writing – review and editing. **Simon Stanworth:** investigation, writing – original draft, writing – review and editing, methodology, supervision, resources, funding acquisition. **Robbie Foy:** conceptualisation, investigation, funding acquisition, writing – original draft, writing – review and editing, methodology, validation, formal analysis, data curation, supervision.

## Ethics Statement

Ethical approval was waived by the research governance, ethics and assurance team, University of Oxford.

## Consent

The authors have nothing to report.

## Conflicts of Interest

The authors declare no conflicts of interest.

## Supporting information

SUPPLE_1.

## Data Availability

Data available on request from the authors.
