# Supplementary material for: Defining Patient and Public Involvement and Engagement Tasks in Health Data Research: A Consensus Study
Source: Health Expect. 2026 Feb 11;29(1):e70578. doi: 10.1111/hex.70578 (PMC12892401; doi:10.1111/hex.70578)
Supplement: Supplementary file 1 — SUPPLE_1. [file HEX-29-e70578-s001.DOC]

**Supplementary Table 1. PPIE tasks with examples**

| 1. ***Prioritising and commissioning research*** 2. **Suggesting research topics**   *For example, people with lived experience of a condition and their carers can suggest areas to look at for specific health conditions in Priority Setting Partnerships (see below) and can suggest new topics for research such as to the National Institute for Health and Care Research (NIHR),* [https://www.nihr.ac.uk/patients-carers-and-the-public/i-want-to-help-with-research/suggest-a-research-topic.htm](about:blank)^[[1]](#footnote-1)^   1. **Setting research priorities for specific health conditions**   *For example, a Priority Setting Partnership between patients, blood donors, carers and clinicians identified and prioritised the most urgent unanswered questions about blood donation and transfusion for future research to address:* <https://www.jla.nihr.ac.uk/priority-setting-partnerships/blood-transfusion-and-blood-donation/>^[[2]](#footnote-2)^ *Later, NIHR launched a funding call for Blood and Transplant Research Units to start addressing these priorities.*   1. **Making recommendations which research to fund**   *For example, patients, donors and carers reviewed funding applications for Blood and Transplant Research Units on NIHR funding committees and recommended which applications to fund,* [*https://www.nihr.ac.uk/committees/*](about:blank)*^[[3]](#footnote-3)^*   1. **Giving views on direction and progress of funded research projects**   *For example, patients and public members adding their perspectives on progress and future plans for funded research as independent data access, oversight or steering committee members.* |
| --- |
| 1. ***Planning research projects*** 2. **Giving feedback on draft funding applications for research**   *For example, patients advised the research team to write the funding application as a co-applicant taking on a specific role in the research team or gave input on specific parts of the application including but not limited to creating an easy-to-understand summary, guiding on acceptability of study or helping to develop Patient and Public Involvement and Engagement plans.*   1. **Helping to shape research questions**   *For example, patients, blood donors and carers worked with clinicians and researchers to include questions they see as important in the development of research projects such as questions addressing quality-of-life.*   1. **Giving views on how the research team will collect and analyse information**   *For example, patients and carers created a survey and advised on questions and wording, who is asked to complete it and how the information is going to be used.*   1. **Guiding the development of interventions including treatments or ways to provide health care**   For example, patients and public members added their perspectives how computerised prompts for GPs may help or hinder communication with patients during consultations.   1. **Advising on ethical, cultural, faith and other issues relating to the research and how to address them**   *For example, patients expressed concerns about lack of ethnicity data that could lead to increasing inequalities which now was included in the project.*   1. **Reviewing plans to recruit participants**   *For example, patients suggested how to ask for consent and how to explain the collection and use of information about ethnicity.*   1. **Reviewing public-facing information about the research to encourage trustworthiness and transparency**   *For example, patients, carers and public members advised and reviewed information explaining research to a public audience and specific underrepresented groups.*   1. **Guiding Patient and Public Involvement and Engagement plans**   *For example, patient and public members planed and co-delivered topics in trainings and meetings for researchers and patients and public members involved.* |
| 1. ***Delivering research*** 2. **Helping to write the research materials**   *For example, patients reviewed research information, which helped health professionals to better explain why research asks about ethnicity and how this data is used.*   1. **Reviewing documents about research for patient and public members**   *For example, patients and carers reviewed participant information to make sure it is clear, easy to understand and addresses important questions such as explaining how patient health data is collected, stored and used.*   1. **Supporting the delivery of Patient and Public Involvement and Engagement plans**   *For example, a public member advised how to best engage with an underserved communities about research and co-facilitated discussions about concerns using health information for patients, carers and public members within their community.*   1. **Encouraging and supporting the involvement of people with diverse views**   *For example, a public member supported outreach, identified barriers and good practice for involvement within their community.*   1. **Helping to build connections to patient networks and communities**   *For example, a community organisation representative invited the research team and advised how to start the discussion with a topic of interst to the community.*   1. **Monitoring the protection of privacy and confidentiality of patient information**   *For example, a patient partner was involved in discussions how information is used safely by research team members and flagged any questions that came up from a patient and public perspective.*   1. **Reviewing progress of research**   *For example, patient representatives received regular updates on the research progress and actively engaged in discussions with the research team.* |
| 1. ***Interpreting research*** 2. **Assisting the research team to interpret research results and make conclusions**   *For example, patient and public members interpreted the results differently to researchers. While researchers saw results as negative, patient and public partners felt results were encouraging,* [*https://www.cancerresearchuk.org/sites/default/files/clinical_case_study_01.pdf*](https://www.cancerresearchuk.org/sites/default/files/clinical_case_study_01.pdf)*^[[4]](#footnote-4)^*   1. **Advising on how to turn results into improved treatments or health care services**   *For example, patients and carers advised communications to the target population about blood iron level screening before larger planned surgery such as a hip replacement.* |
| 1. ***Sharing and using research knowledge*** 2. **Sharing knowledge from the research**   *For example, patients helped writing publications and Plain English summaries as co-authors.*   1. **Advising how to feedback research results to participants**   *For example, patient representatives worked with the research team to thank participants, helped create accessible formats of study results and identified ways to reach as many people as possible.*   1. **Adding personal perspectives to research results**   *For example, patients, carers, and blood donors added a personal perspective how they see the research may help someone in a similar situation in future and their lessons from being involved in research.*   1. **Speaking about research results and point out groups to discuss it with**   *For example, patients (co-)presented study results at conferences, to patient networks and/or community groups.*   1. **Giving personal insights into how patients and public members see study results**   *For example, patients worked with the research team to explain benefits of their research project for specific, local communities.* |
| 1. ***Evaluating research*** 2. **Contributing to the evaluation of research project**   *For example, patients, carers and public members gave feedback on areas that can be done differently next time such as recruitment or communication about research.*   1. **Helping to evaluate the impact of Patient and Public Involvement and Engagement**   *For example, patients, carers and public members provided feedback on the experience and learnings being involved in research and wrote case studies with the research team what has changed as a result of their input.*   1. **Making future research recommendations**   *For example, patients and carers helped to remain focused on the issues for people with lived experience of a condition or service such as medical support tools to screen for low blood iron levels well as too high iron levels before planned surgery.* |

**References**

1. **Suggest new research topics to the National Institute for Health and Care Research** *(NIHR)*

<https://www.nihr.ac.uk/patients-carers-and-the-public/i-want-to-help-with-research/suggest-a-research-topic.htm>

1. **NHS Blood and Transplant and NIHR** [**Oxford Biomedical Research Centre**](about:blank) **Priority Setting Partnership**

<https://www.jla.nihr.ac.uk/priority-setting-partnerships/blood-transfusion-and-blood-donation/>

1. **NIHR funding committees recommending which applications to fund** [*https://www.nihr.ac.uk/committees/*](about:blank)
2. **Researchers and people with lived experience may view the findings differently**

[*https://www.cancerresearchuk.org/sites/default/files/clinical_case_study_01.pdf*](https://www.cancerresearchuk.org/sites/default/files/clinical_case_study_01.pdf)

Supplementary Table 1: 29 Patient and Public Involvement and Engagement (PPIE) tasks across the research cycle with examples.

1. See references for content of hyperlinks [↑](#footnote-ref-1)
2. [↑](#footnote-ref-2)
3. See references for content of hyperlinks [↑](#footnote-ref-3)
4. See references for hyperlinks [↑](#footnote-ref-4)
